# How User Characteristics Affect Use Patterns in Web-Based Illness Management Support for Patients with Breast and Prostate Cancer

**DOI:** 10.2196/jmir.2285

**Published:** 2013-03-01

**Authors:** Elin Børøsund, Milada Cvancarova, Mirjam Ekstedt, Shirley M Moore, Cornelia M Ruland

**Affiliations:** ^1^Centre for Shared Decision Making and Collaborative Care ResearchOslo University HospitalOsloNorway; ^2^Royal Institute of Technology, KTHSchool of Technology and HealthStockholmSweden; ^3^Frances Payne Bolton School of NursingCase Western Reserve UniversityCleveland, OHUnited States; ^4^Department of MedicineUniversity of OsloOsloNorway

**Keywords:** Web-based intervention, Internet, symptom management, self-care, use patterns, user characteristics, targeting

## Abstract

**Background:**

Frequently eHealth applications are not used as intended and they have high attrition rates; therefore, a better understanding of patients’ need for support is warranted. Specifically, more research is needed to identify which system components target different patient groups and under what conditions.

**Objective:**

To explore user characteristics associated with the use of different system components of a Web-based illness management support system for cancer patients (WebChoice).

**Methods:**

For this secondary post hoc analysis of a large randomized controlled trial (RCT), in which WebChoice was tested among 325 breast cancer and prostate cancer patients who were followed with repeated measures for 1 year, usage patterns of 162 cancer patients in the intervention arm with access to WebChoice were extracted from the user log. Logistic regression was performed to identify patterns of associations between system use and patient characteristics. Latent class analyses (LCA) were performed to identify associations among the use of different system components and levels of social support, symptom distress, depression, self-efficacy, and health-related quality of life.

**Results:**

Approximately two-thirds (103/162, 63.6%) of the patients logged on to WebChoice more than once, and were defined as users. A high level of computer experience (odds ratio [OR] 3.77, 95% CI 1.20-11.91) and not having other illnesses in addition to cancer (OR 2.10, 95% CI 1.02-4.34) increased the overall probability of using WebChoice. LCA showed that both men with prostate cancer and women with breast cancer who had low scores on social support accompanied with high levels of symptom distress and high levels of depression were more likely to use the e-message component. For men with prostate cancer, these variables were also associated with high use of the self-management advice component. We found important differences between men with prostate cancer and women with breast cancer when associations between WebChoice use and each user characteristic were analyzed separately. High use of all components was associated with low levels of social support among women with breast cancer, but not among men with prostate cancer. High use of e-messages, advice, and the discussion forum were associated with high levels of depression among women with breast cancer, but not among men with prostate cancer. For men with prostate cancer (but not women with breast cancer), high use of symptom assessments, advice, and the discussion forum were associated with high levels of symptom distress. However, it is unclear whether these findings can be attributed to differences related to diagnosis, gender, or both.

**Conclusions:**

This study provides evidence that different user characteristics are associated with different use patterns. Such information is crucial to target Web-based support systems to different patient groups. LCA is a useful technique to identify subgroups of users. In our study, e-messages and self-management advice were highly used components for patients who had low levels of social support and high illness burden, suggesting that patients with these characteristics may find such tools particularly useful.

**Trial Registration:**

ClinicalTrials.gov NCT00710658; http://clinicaltrials.gov/ct2/show/NCT00710658 (Archived by WebCite at http://www.webcitation.org/6EmEWZiwz)

## Introduction

Web technologies provide an opportunity to target support to different patient groups and can reach a large number of users. Recent years have seen growth in Web-based interventions and support systems that have been shown to assist a wide range of patients successfully [1-6], including people with cancer [7-9], asthma [10], arthritis [11], and heart disease [12]. They have also been found to assist in health behavior change, such as improving diet and weight loss [13], increasing physical activity [14], or in smoking cessation [15]. According to a Cochrane review of 24 randomized clinical trials that summarized the effects of different Web-based interventions for people with chronic diseases, these interventions had a significant effect on knowledge, perceived social support, health behaviors, clinical outcomes, and a possible positive effect on self-efficacy [2].

Web-based interventions that provide targeted support are more likely to be successful because information that is relevant to specific groups is more likely to be used [16]. However, successful targeting presents challenges because characteristics of the user groups of Web-based interventions are not clear. Recently the Comprehensive Model of Information Seeking (CMIS) was developed to better understand how user characteristics affect the use of Web-based interventions [17]. The model includes antecedent factors (demographics, personal experiences, salience, and beliefs), information carrier factors (characteristics and utility of the information channels), and information-seeking actions. The CMIS has recently been used as a framework to understand use of an interactive cancer communication system [18,19].

Demographic factors are considered important in predicting the use of health information resources in the CMIS framework, and are reported to influence use in several eHealth studies. Older age [20-23], female gender [20,24,25], higher education [20,21,24-28], and higher income [27,29] have all been associated with higher use in some studies, whereas other studies show younger age [26,28,29] to be associated with higher use. A recent systematic review of patients’ acceptance of health information technologies revealed no consistent effect of age or gender on acceptance [30]. However, acceptance increased with higher education. On the other hand, level of education did not influence use of a Web-based diabetes program, and neither did age or health literacy [31]. These divergent findings might reflect ongoing change in Internet sociodemographics and dynamics [20,32].

According to CMIS, a person’s direct experience with a disease will affect their need for information and predict their health-seeking behavior [17]. Different diagnoses cause different symptoms, need different treatment, and have different illness trajectories. Higher levels of functional well-being [33] and not having a chronic condition [22] are also associated with higher use of eHealth applications.

Psychosocial factors also affect information-seeking behaviors. For example, a person’s health beliefs and perceived salience of the information affect information seeking [17], as does the individual’s perception of their ability to control events. This could go both ways. A person with high self-efficacy might have higher confidence in seeking and using information in eHealth applications. On the other hand, for a person with low self-efficacy, eHealth applications could be an additional tool improving his or her confidence or capacity to deal with their symptoms and treatment [34]. The level of social support could affect how much information and support is needed. Lower levels of social support and symptoms of depression or negative mood have also been associated with higher use of eHealth applications [18,24,33,34]. In addition, prior Internet experience has been identified as a factor linked with increased use and acceptance of eHealth applications in some studies [23,30], but not others [31].

Although eHealth applications have been shown to be effective and can offer easier communication and cost savings, high rates of dropout and nonusage have been shown in many eHealth studies [35,36]. Moreover, users tend to use these applications differently than intended, indicating a need for a better understanding of patients’ varying needs for support and for examining the best way to deliver eHealth applications. Research aiming to identify which components can be the most beneficial for different patient groups is critical in optimizing such systems to patients’ preferences [2,35,37]. Because Web-based support systems usually include more than one component, it is not known yet which components may be particularly helpful to patients. Also, patients may prefer different types of support features, and their support needs may vary based on type of illness or user characteristics. Furthermore, although perceptions of a system’s perceived usefulness have been investigated in a number of studies, the systems have primarily been evaluated as a whole on a set of general criteria, and the usefulness of specific components that the system offers have not been addressed. An average usefulness score in these types of studies may well result from a user who has evaluated some aspects as high and some as low. To design Internet systems that can better target different user groups, more research is needed examining which user group characteristics are associated with different types and use of Internet support [2]. Exploring the characteristics of different users is of importance in refining and optimizing Web-based interventions to better fit different patient groups and increase their potential advantages.

The aim of this exploratory study was to describe user characteristics associated with the use of different components of WebChoice [7], a Web-based illness management support system for cancer patients. The following research questions were addressed:

1. What demographic-related, illness-related, and psychosocial variables are associated with the use of WebChoice?

2. Among WebChoice users, what are the associations among levels of patients’ symptom distress, social support, depression, self-efficacy, health-related quality of life, and their use of different WebChoice components?

## Methods

This exploratory study is a post hoc analysis of a large randomized controlled trial (RCT) in which WebChoice was tested among 325 breast cancer and prostate cancer patients who were followed with repeated measures for 1 year [7]. In the RCT, effects on symptom distress, depression, self-efficacy, health-related quality of life (HRQOL), and social support were measured. Use of WebChoice significantly reduced symptom distress, and patients in the WebChoice group also showed significant within-group improvements in depression during the study period. This was not observed in the control group; the control group also experienced significant deterioration of self-efficacy and HRQOL [7].

Recruitment took place between May 2006 and July 2007. Patients were recruited through advertisements in newspapers, on the Norwegian Cancer Society’s website and in their magazine, and through information pamphlets mailed to patients through the Norwegian Cancer Registry. Patients who were interested called the research center to participate. Inclusion criteria were age over 18 years, able to read/speak Norwegian, having Internet access at home, and undergoing active treatment for breast or prostate cancer.

In total, 325 cancer patients took part in this study. Patients in the experimental group (n=162) who had access to WebChoice for 1 year constituted the sample for this paper. Participants received a user manual for WebChoice and a smart card-based public key solution for secure system access. They were instructed that they could use the system as much or as little as they liked. All data were submitted to a secure server using an encrypted connection. The study was approved by the Regional Committee for Medical and Health Research Ethics and the Data Security Inspectorate in Norway. Written informed consent was obtained from all participants. WebChoice could be used with both slow and fast Internet connections. Download times for the different components were the same.

### Intervention

**Figure 1:**
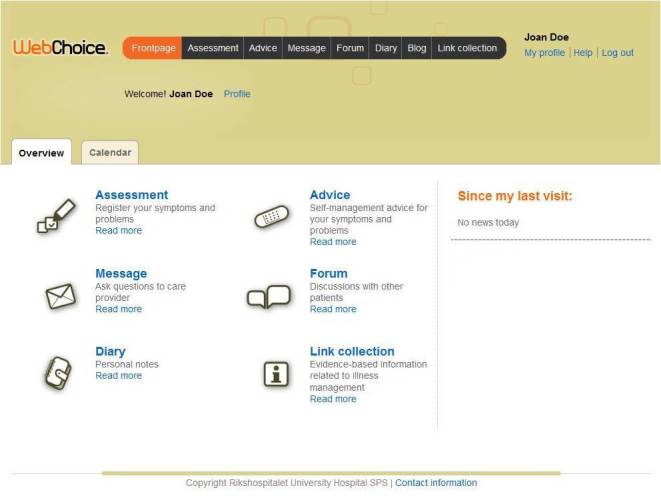
Screenshot of the WebChoice overview page.

**Figure 2:**
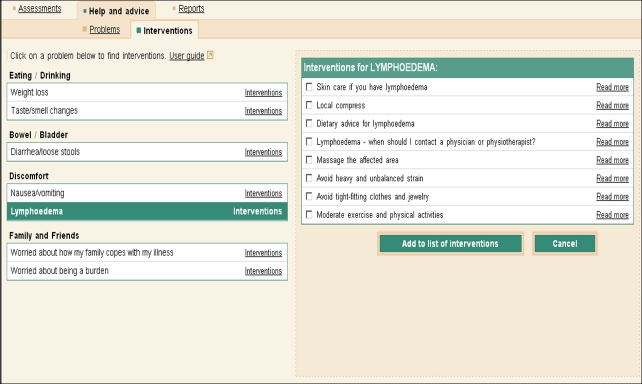
Screenshot of the results of an assessment and the associated advice/interventions.

**Figure 3:**
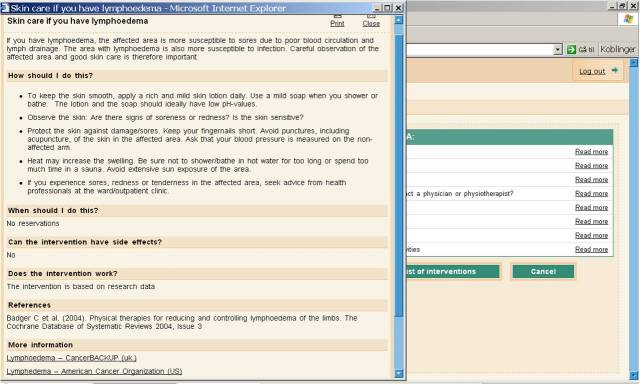
Screenshot showing an example of content and layout in the advice component.

WebChoice [38] was developed in close cooperation with users and health care personnel [39]. The modules tested in this study targeted breast and prostate cancer patients and contained the following components (see Figure 1):

1. An assessment component in which patients could monitor their symptoms, problems, and priorities for support in physical, functional, and psychosocial dimensions. Patients choose symptoms and problems they were experiencing from a predefined list, and could rate the burden of these and what they needed help with. This information could be used to monitor improvement/deterioration of the condition, knowing when to alert health care personnel, preparing for a hospital/physician consultation, improving patient-provider communication, or with obtaining immediate access to self-management advice components described subsequently (Figure 2).

2. An advice component provided illness self-management support. Patients’ self-reported symptoms/problems triggered the display of appropriate self-management activities that patients could choose from to relieve their symptoms and problems (Figure 2). Each choice contained an explanation of what the activity was; how to perform it; potential risks, side effects, and contraindications; when to contact a physician; levels of evidence; references to the source of the evidence; and links to other reliable websites for related information (Figure 3).

3. An information component in which patients had access to other reliable Web sources in Norwegian and English, such as information about tests, treatments, and potential side effects, lifestyle suggestions, and information about patients’ legal rights.

4. A communication component for sharing experiences with other patients or for obtaining help from oncology nurses. Patients could participate in an online forum group discussion that allowed them to exchange messages anonymously with other patients, or use the online messaging system for private e-communication in which they could ask questions, share experiences, and get advice from oncology nurses. The nurses in this study were employed at the research center and were not involved in the direct care of the patients. They logged onto the communication component each weekday and contributed to the discussion group when appropriate.

5. An electronic diary in which patients could keep personal notes.

### Measures of System Use

Data on system use were extracted from the user logs on the server. Those who had logged on 0 to 1 times were categorized as nonusers; those who had logged on twice or more were categorized as users. We specified a minimum of 2 log-ins because patients who only logged on once may have only read the welcome message and never actually used the system. Information was collected on how many times the users logged on, how much time they spent on the site and on each component, and which components of WebChoice were accessed or used actively.

**Table 1:** Measures of system use of the WebChoice website.

Variable	Description
Total visits	Total number of times that a user logged on to the system.
Duration	Minutes spent using the system and its different components. If a user, when visiting a component, did not make a Web server request within the 20 minutes, the visit was ended. After the last Web server request during the visit, 10 minutes were added to the duration of this visit to reflect the fact that most usage consisted of reading information (an activity that cannot be logged). Duration is a complicated measure because of a lack of control over what the user actually does while logged on, and because some users do not log out of the system after visiting a component.
Assessments	Number of times a user chose symptoms and problems from a predefined list and generated their own symptom list.
Messages sent	Number of messages that users sent to the oncology nurse.
Posts in forum	Number of postings that users made in the discussion forum.
Diary notes	Number of notes made in the diary.
Visits to the different components	Number of times that a user entered a component. Unlike the previous measures, this measure does not indicate whether or not an action was taken within the component, for example, participants could visit a component to read what they had written previously or what others had written (eg, forum or answers from nurses).

### User Characteristics

Demographic variables (age, gender, marital status, level of education, and household income) and diagnosis-related variables (diagnosis of breast or prostate cancer, time since diagnosis, recurrence/metastasis, type of treatment, and other illnesses) were recorded with a study-specific questionnaire.

Symptom distress was measured by using the 32-item Memorial Symptom Assessment Scale-Short Form (MSAS-SF) [40]. Symptom distress was measured with 5-point Likert scales, in which respondents rated the degree from 0 (not at all) to 4 (very much). Higher scores indicated greater symptom distress. Cronbach alpha for our sample at baseline was .92.

Social support was measured with the 20-item Medical Outcomes Study Social Support Survey (MOS-SS), including 5 subscales addressing emotional, instrumental, tangible, and affectionate support, and positive social interaction [41]. Responses on 5-point Likert scales ranged from 0 (none of the time) to 4 (all the time). Higher scores indicated more social support. Cronbach alpha for our sample at baseline was .96.

Depression was measured with the 20-item Center for Epidemiological Studies Depression scale (CES-D) [42] with responses on 4-point Likert scales ranging from 0 (rarely or none of the time) to 3 (most or all the time). Higher scores indicated greater depression. Cronbach alpha for our sample at baseline was .88.

Self-efficacy was measured with the 33-item Cancer Behavior Inventory (CBI) version 2.0 [43] which measured coping self-efficacy with cancer-related stress on 7 dimensions: (1) maintenance of activity and independence, (2) seeking and understanding medical information, (3) stress management, (4) coping with treatment-related side effects, (5) accepting cancer and maintaining a positive attitude, (6) affective regulation, and (7) seeking support. Responses on 9-point Likert scales ranged from 1 (not at all confident) to 9 (totally confident). Higher scores indicated greater self-efficacy. Cronbach alpha for our sample at baseline was .95.

HRQOL was measured using the 15-item 15D preference-based single index [44]. From 5 ordinal levels on each dimension, respondents chose the one that best described their present health status. Higher scores indicated greater HRQOL. Cronbach alpha for our sample at baseline was .77.

Computer experience was assessed with a simple question asking for patients’ experience with computer use, ranging from 1 (no computer experience at all) to 5 (a lot of computer experience).

To prepare the data for latent class analysis (LCA), total scores on symptom distress, social support depression, self-efficacy, HRQOL, and the data on system use of the different WebChoice components were divided into tertiles based on the scores for breast and prostate cancer patients combined, using the whole sample of patients with access to WebChoice (Multimedia Appendix 1).

Marital status was dichotomized as married/cohabitating and any other status as single. Education was simplified from 4 to 3 categories. Elementary and high school were merged because there were few participants in the “elementary school only” group. Income was recoded from 5 to 3 categories. Computer experience was recoded from 5 to 3 categories because the 2 lowest ranks were related to no or little experience and the 2 highest described a great deal of computer experience.

### Statistical Analyses

Data are presented as medians and interquartile ranges (IQRs) for continuous variables and as proportions for categorical data. Associations between categorical variables among users and nonusers were analyzed using the chi-square test for pairs of categorical variables; the Mann-Whitney test was used for continuous data with skewed distributions. A multiple logistic regression model was fitted to compare users and nonusers on demographic, disease-related, and psychosocial variables. Variables with a *P* value <.2 in the univariate logistic regression analyses were included in a multiple regression model. Based on the assumption that we could not expect a linear association between age and the outcome, age was first divided into several categories. Later, categories with similar odds ratios (ORs) were combined resulting in a dichotomizing of the age variable.

To identify characteristics associated with use of different components of WebChoice among users, we used LCA, which is a statistical method designed to identify if there are underlying types or subgroups of individuals that share specific characteristics [45]. LCA can best be thought of as an improved cluster analysis. It is a pattern recognition technique based on the statistical concept of likelihood and, thus, based on the same principle as factor analysis. The main difference is that cases are not absolutely assigned to classes, but have a probability of membership for each class. The results are presented with estimated probabilities and formal statistical tests can be performed to evaluate different models. The main goal for this LCA was to identify how a set of user-related variables could be associated with different system use. Utilizing LCA made us not only able to identify these associations, but also to quantify their direction and strength. Because of a limited sample size, we fitted LCA models with 3 classes and 4 explanatory variables at most to avoid overfitting and multicollinearity. As a final step, the best models were chosen based on the Akaike information criterion (AIC) [46] and the Bayesian information criterion (BIC) [47].

We expected different user patterns for breast and prostate cancer patients because these patient groups differed with regard to gender, age, treatment, and presence of other illnesses. Two-thirds (37/56) of the breast cancer patients were treated with chemotherapy compared to 26% (12/47) of the prostate cancer patients. Therefore, all LCA models were stratified by type of diagnosis and adjusted for age at inclusion. The cutoffs used to categorize variables were based on a tertile division of the entire sample. Because the division was based on the actual data and not on predefined cutoffs, a tertile division was chosen to provide categories with sufficient sizes and enable us to make distinctions among the high, medium, and low values. To ensure a sufficient number of breast and prostate cancer patients within each tertile-based category, patient numbers were checked for each variable (Multimedia Appendix 1) and the numbers were found to be satisfactory.

To select variables for inclusion in the final LCA models, we first fitted models in which 1 psychosocial variable at a time was tested together with 3 user variables. The psychosocial variables that revealed clear patterns of use were kept and integrated in the final LCA models, where a cluster of psychosocial variables was tested with single user variables.

The descriptive statistics and logistic regression were carried out using SPSS version 16.0 (SPSS Inc, Chicago, IL, USA). LCA was performed with SAS version 9.3 (SAS Institute Inc, Cary, NC, USA), by using the PROC LCA procedure for LCA [48]. All tests were 2-sided and *P* values <.05 were considered statistically significant.

## Results

Of the 162 participants who had access to WebChoice, 103 (63.6%) logged on twice or more over the 1-year study period and were defined as *users* (Table 2). There were no statistically significant differences in demographic, disease-related, or psychosocial variables between users and nonusers of WebChoice (Tables 2 and 3). Although not statistically significant, there were indications of higher use among men with prostate cancer (*P*=.09), patients without other illnesses (*P*=.08), and patients with more computer experience (*P*=.07) (see Table 2).

### User Characteristics Associated with Use of WebChoice

Baseline scores on symptom distress, social support, depression, HRQOL, and self-efficacy did not differ significantly between users and nonusers; therefore, they were not included as covariates in the logistic regression models. Multiple logistic regressions revealed that high levels of computer experience (OR 3.77, 95% CI 1.20-11.91) and not having other illnesses (OR 2.10, 95% CI 1.02-4.34) were significantly associated with increased use of WebChoice after controlling for type of diagnosis and age (Table 4). The other illnesses most frequently reported were heart disease, rheumatic illness, lung disease, muscle and skeletal conditions, diabetes, and neurologic conditions.

### Frequencies of Use of Different WebChoice Components

As displayed in Table 5, the WebChoice components visited 1 or more times by most of the users during the year of study participation were the advice component (98/103, 95.1%), the information component (96/103, 93.2%), assessments (95/103, 92.2%), and messages (93/103, 90.3%).

Patients visited the discussion forum far more often than they submitted their own postings, and the discussion forum was the component in which the patients spent most time (median 84 minutes, range 0-5108). Similarly, patients visited the message component far more often than they sent messages; the median time spent using this component was 21 minutes (range 0-701). The diary was among the least-used components in WebChoice. There were large differences between users. When we analyzed patterns of use of different components in WebChoice, it became apparent that different users used components quite differently, eg, a participant with high e-message use did not necessarily use the assessments or advice more often. Therefore, as the next step, we analyzed which user characteristics were associated with utilizing specific components.

### Associations Between User Characteristics and Component Use

Overall, we did not find any association among use of different WebChoice components (measured in minutes) and demographics, most disease-related characteristics, or computer experience.

However, use patterns were different for men with prostate cancer and women with breast cancer. Levels of use were related to several psychosocial factors. When fitting separate models for 1 user characteristic at a time together with 3 WebChoice components, some important differences emerged (Table 6). Degree of social support and depression were more important for overall WebChoice use for women with breast cancer, whereas symptom distress was more influential for men with prostate cancer. High use of all WebChoice components was associated with low levels of social support among women with breast cancer. No such pattern was detected for men with prostate cancer. High use of advice, e-messages, and the discussion forum was associated with high levels of depression in women with breast cancer, but not in men with prostate cancer. For men with prostate cancer, high use of symptom assessments, advice, and the discussion forum was associated with high levels of symptom distress. Symptom distress did not appear to impact the use of WebChoice for women with breast cancer. No specific patterns of use were associated with levels of self-efficacy or HRQOL. Different combinations of patients’ levels of social support, symptom distress, depression, and use of the components were explored with LCA. Because HRQOL and self-efficacy were not clearly associated with use, these variables were not included in the final LCA models. A summary of associations among patients’ characteristics and selected components of WebChoice can be found in Table 6.

#### Use of the Message Component

The LCA revealed that lower levels of social support, higher levels of symptom distress, and higher depression were associated with higher use of messages to oncology nurses. This applied to both men with prostate cancer and women with breast cancer (Table 7). The estimated probabilities in Table 6 can be interpreted as follows: the model has identified a class of patients with breast cancer, latent class 1. Members of latent class 1 have a .60 probability of high use of messages, a .96 probability of low social support, a .74 probability of high symptom distress, and a .89 probability of depression.

#### Use of the Advice Component

Lower levels of social support, higher levels of symptom distress, and higher levels of depression were associated with higher use of the advice component among men with prostate cancer, but we did not detect such an association among women with breast cancer (Table 8).

#### Use of the Discussion Forum

Analyses of the discussion forum section revealed that higher levels of social support and lower level of symptom distress and depression among men with prostate cancer were associated with low use of the forum (Table 9). No clear pattern was detected among women with breast cancer.

No clear patterns of use associated with any user characteristics were found for the level of minutes spent at the assessment and information components.

**Table 2:** Characteristics of all breast and prostate cancer patients (users and nonusers) with access to WebChoice.

Characteristics			WebChoice access N=162	Users n=103	Nonusers n=59	*P* value
**Demographic factors**						
	**Age, median (range)**		57 (35-80)	58 (36-79)	56 (35-80)	.15
	**Marital status, n (%)**					.94
		Married/cohabitating	135 (83.3)	86 (83.5)	49 (83.1)	
		Single/divorced	27 (16.7)	17 (16.5)	10 (16.9)	
	**Education, n (%)**					.89
		Elementary/high school	62 (38.3)	38 (36.9)	24 (40.7)	
		University/college ≤4 years	69 (42.6)	45 (43.7)	24 (40.7)	
		University/college >4 years	31 (19.1)	20 (19.4)	11 (18.6)	
	**Household annual income (NOK), n (%)** ^**a**^					.63
		<400,000	48 (29.6)	32 (31.1)	16 (27.1)	
		400,000 to 600,000	44 (27.2)	26 (25.2)	18 (30.5)	
		>600,000	65 (40.1)	44 (42.7)	21 (35.6)	
		Missing data	5 (3.1)	1 (1.0)	4 (6.8)	
**Disease-related factors**						
	**Diagnosis, n (%)**					.09
		Breast cancer	96 (59.3)	56 (54.4)	40 (67.8)	
		Prostate cancer	66 (40.7)	47 (45.6)	19 (32.2)	
	**Months since diagnosis, median (IQR)^b^**		11.5 (21)	11.0 (22)	12.0 (20.3)	.52
	**Metastasis, n (%)**		26 (16.0)	18 (17.5)	8 (13.6)	.51
	**Recurrence, n (%)**		13 (8.0)	6 (5.8)	7 (11.9)	.17
	**Other illnesses, n (%)**		63 (38.9)	35 (34.0)	28 (47.5)	.08
**Psychosocial factors, median (IQR)** ^**b**^						
		Symptom distress	28 (27)	29 (28)	25 (23)	.49
		Social support	84 (28)	84 (28)	84 (31)	.38
		Depression	9.5 (12)	10 (12)	9 (12)	.51
		Self-efficacy	219 (67)	216 (71)	227 (64)	.57
		Health-related quality of life	0.86 (0.16)	0.86 (0.15)	0.88 (0.16)	.49
	**Computer experience, n (%)**					.07
		None/little	16 (9.9)	6 (5.8)	10 (16.9)	
		Medium	48 (29.6)	32 (31.1)	16 (27.1)	
		High	93 (57.4)	62 (60.2)	31 (52.5)	
		Missing data	5 (3.1)	3 (2.9)	2 (3.4)	

^a^ NOK = Norwegian kroner (400,000 NOK≈US $67,000; 600,000 NOK≈US $100,000).

^b^ IQR = Interquartile range.

**Table 3:** Baseline treatment characteristics of patients with access to WebChoice by diagnosis.

Diagnosis and treatment		WebChoice access n (%)	Users n (%)	Nonusers n (%)	*P* value
**Breast cancer (n=96)** ^**a**^					
	Radiotherapy	62 (65)	37 (66)	25 (63)	.72
	Chemotherapy	71 (74)	40 (71)	31 (78)	.50
	Hormone treatment	61 (64)	37 (66)	24 (60)	.54
**Prostate cancer (n=66)** ^**a**^					
	Radiotherapy	17 (26)	12 (26)	5 (26)	.95
	Chemotherapy	4 (6)	3 (6)	1 (5)	.99
	Hormone treatment	41 (62)	26 (74)	15 (88)	.30

^a^ Patients can be given several treatments simultaneously.

**Table 4:** Binary logistic regression of patient characteristics and use of WebChoice (2 or more log-ins) (N=162).

Sociodemographic and health characteristics		Univariate analysis			Multiple analysis		
		OR^a^	95% CI	*P* value	OR^a^	95% CI	*P* value
**Diagnosis**							
	Breast cancer (ref)	1.00					
	Prostate cancer	1.77	0.90-3.45	.10	1.45	0.65-3.23	.36
**Age**							
	<50 years (ref)	1.00					
	≥50 years	1.69	0.83-3.42	.15	1.75	0.74-4.13	.20
**Other illnesses**							
	Yes (ref)	1.00					
	No	1.79	0.93-3.45	.08	2.10	1.02-4.34	.045
**Computer experience**							
	Low (ref)	1.00					
	Medium	3.33	1.02-10.81	.05	3.09	0.91-10.49	.07
	High	3.33	1.11-10.02	.03	3.77	1.20-11.91	.02

^a^OR: odds ratio.

**Table 5:** Usage of different components of WebChoice over the year of accessibility (N=103).

Components in WebChoice	Times accessed			Users who accessed at least once	
	Median	IQR^a^	Range	n	%
Total visits	12	29	2-892	103	100
Total duration (minutes)	250	490	21-11,167	103	100
Assessments	2	5	0-51	77	74.8
Assessment visits	7	17	0-103	95	92.2
Assessment duration (minutes)	13	33	0-254	95	92.2
Advice visits	5	9	0-63	98	95.1
Advice duration (minutes)	15	32	0-372	98	95.1
Information section visits	4	7	0-97	96	93.2
Information duration (minutes)	25	54	0-431	96	93.2
Messages sent	1	5	0-49	62	60.2
Total messages visits	6	13	0-163	93	90.3
Message duration (minutes)	21	68	0-701	93	90.3
Posts in forum	0	4	0-58	50	48.5
Forum visits	8	36	0-536	87	84.5
Forum duration (minutes)	84	309	0-5108	87	84.5
Diary notes	1	4	0-142	54	52.4
Diary visits	2	6	0-94	73	70.9
Diary duration (minutes)	2	32	0-1003	73	70.9

^a^ IQR=interquartile range.

**Table 6:** Summary of associations among single patient characteristics or a cluster of patients’ characteristics and use of components in WebChoice stratified by diagnosis.

Characteristics		Associations with use of components in WebChoice	
		Prostate cancer	Breast cancer
**Single characteristics** ^**a**^			
	Low social support	No associations	High use of assessment, advice, information, messages, and forum
	High symptom distress	High use of assessments, advice and forum	No associations
	High depression	No associations	High use of advice, messages, and forum
	Low health-related quality of life	No associations	No associations
	Low self-efficacy	No associations	No associations
**Cluster of characteristics** ^**b**^			
	Low social support, high levels of symptom distress and high levels of depression	High use of messages and advice	High use of messages

^a^ Tables with exact values for the probability of use of different components based on single patient characteristics can be found in Multimedia Appendix 2.

^b^ Tables with exact values for the probability of use of different components based on a cluster of characteristics can be found in Tables 7-9.

**Table 7:** Use of the message component in WebChoice (in minutes). Latent class model, association with levels of social support, symptom distress, and depression. The numbers represent item probabilities. All models were stratified by diagnosis and adjusted for age at inclusion.

Variables		Prostate cancer latent class^a^			Breast cancer latent class^a^		
		1^b^	2	3	1^b^	2	3
**Use of messages**							
	Low	.33	.37	.44	.20	.23	.45
	Medium	.09	.32	.27	.20	*.76*	.07
	High	*.57*	.31	.28	*.60*	.01	.48
**Social support**							
	Low	*.63*	.09	.11	*.96*	.21	.26
	Medium	.10	*.61*	.26	.03	*.52*	.41
	High	.27	.30	*.63*	.02	.27	.32
**Symptom distress**							
	High	*.51*	.36	.11	*.74*	.51	.01
	Medium	.24	.38	.26	.24	.13	*.56*
	Low	.25	.26	*.63*	.01	.36	.43
**Depression**							
	High	*.87*	.01	.01	*.89*	.26	.07
	Medium	.12	*.97*	.02	.10	.47	.36
	Low	.02	.02	*.97*	.01	.27	*.57*

^a^ Item response probabilities >.5 in italics to facilitate interpretation.

^b^ Most prominent class.

**Table 8:** Use of the advice component in WebChoice (in minutes). Latent class model, association with levels of social support, symptom distress, and depression. The numbers represent item probabilities. All models were stratified by diagnosis and adjusted for age at inclusion.

Variables		Prostate cancer latent class^a^			Breast cancer latent class^a^		
		1^b^	2	3	1	2	3
**Use of advice**							
	Low	.02	*.50*	.15	.06	.18	*.65*
	Medium	.19	.25	*.83*	*.51*	.18	.21
	High	*.80*	.25	.03	.43	*.64*	.15
**Social support**							
	Low	*.69*	.09	.26	*.92*	.47	.01
	Medium	.14	*.55*	.01	.08	.34	*.59*
	High	.17	.35	*.73*	.01	.19	.40
**Symptom distress**							
	High	*.79*	.29	.02	*.72*	.01	.41
	Medium	.20	.33	.28	.22	.28	.34
	Low	.02	.38	*.70*	.06	*.70*	.25
**Depression**							
	High	*.92*	.01	.29	*.81*	.08	.18
	Medium	.02	*.63*	.01	.19	.18	*.55*
	Low	.06	.37	*.69*	.01	*.74*	.27

^a^ Items response probabilities >.5 in italics to facilitate interpretation.

^b^ Most prominent class.

**Table 9:** Use of the forum component in WebChoice (in minutes). Latent class model, association with levels of social support, symptom distress, and depression. The numbers represent item probabilities. All models were stratified by diagnosis and adjusted for age at inclusion.

Variables		Prostate cancer latent class^a^			Breast cancer latent class^a^		
		1	2	3^b^	1	2	3
**Use of forum**							
	Low	*.57*	.27	*.93*	.09	.35	.15
	Medium	.01	.44	.06	.49	.32	.41
	High	.42	.29	.01	.42	.34	.43
**Social support**							
	Low	*.73*	.11	.01	*.72*	.49	.01
	Medium	.11	*.60*	.14	.23	.32	*.54*
	High	.16	.29	*.85*	.05	.19	.45
**Symptom distress**							
	High	*.55*	.25	.15	*.69*	.02	.33
	Medium	.35	.34	.19	.22	.19	.43
	Low	.10	.40	*.66*	.09	*.79*	.23
**Depression**							
	High	*.73*	.01	.14	*.82*	.02	.02
	Medium	.17	*.70*	.02	.17	.17	*.66*
	Low	.10	.29	*.84*	.01	*.82*	.33

^a^ Items response probabilities >.5 in italics to facilitate interpretation.

^b^ Most prominent class.

## Discussion

In this study, we explored how cancer patients’ demographic, disease-related, and psychosocial factors were associated with the use of different components of a Web-based self-management support system for cancer patients. Men with prostate cancer and women with breast cancer who reported low levels of social support and high levels of symptom distress and depression indicating high illness burden made high use of sending e-messages to oncology nurses. Men with prostate cancer with these characteristics also made high use of the advice component. Levels of social support and depression were more important for use patterns among women with breast cancer, and symptom distress was more influential for men with prostate cancer.

Research studies designed to improve understanding of how different subgroups of patients use Web-based support systems can provide insight into how to target such systems and better meet the needs of user groups. This study makes an important contribution to this area. To our knowledge, this is the first study that systematically evaluated how a cluster of factors, such as social support, symptom distress, and depression, were associated with patients’ use of different system components. Although use does not necessarily reflect usefulness or patients’ preferences for different system components, the study results suggest that there are identifiable subgroups of patients who make different use of Web-based support. It also confirms that there are no “one size fits all” patterns of use or systems of support. Although much more research in this area is needed, our findings hold promise that we may eventually be able to identify specific patient characteristics or preferences through use of appropriate screening tools that will allow us to offer the right set of support components for specific patient groups.

It is acknowledged that behavioral and self-management interventions are more effective when they include more than 1 mechanism for support, but it is challenging to determine which components have the most positive effects. One way to address this question would be to perform RCTs with multiple intervention groups, assigning them different dosages or components of the Web-based support system. For example, Baker and colleagues [49] explored 3 different combinations of an interactive cancer communication system compared with regular Internet access. Results revealed that the information and support services significantly benefited breast cancer patients, but more complex and interactive services did not. Studies with multiple groups need a large sample size to be able to detect clinically relevant group differences, which is costly and not always feasible. Identification of subgroups of users, as in our study, does not predict the relative contribution of different components to achieve positive outcomes, but it can help identify potential candidates for component inclusion in future studies.

### User Characteristics Associated with Patterns of Use

Among those who became users of WebChoice, our study suggests that several factors affected the use of different components. Participants with high symptom distress and depression, indicating high illness burden, and who also had low social support utilized the e-message service and advice component the most. Demographic and other factors did not play a role in their use patterns. High illness burden accompanied with low social support might indicate a higher need for support than for those who do relatively well, and these patients might have potentially more to gain from the e-message component. The analyses of messages presented in a previous paper identified that living with physical symptoms and side effects, living with a fear of relapse, concerns about everyday life, and unmet informational needs from health care providers were important themes in these messages and were used to address both emotional and informational issues by breast and prostate cancer patients alike [50]. Although used by less than two-thirds of users (62/103, 60.2%), the message service was evaluated by patients as a supportive and useful component [51]. This is consistent with findings from a study on a Web-based support system for diabetes patients in which email communication with a nurse was also highlighted as an important reason for using the system [36]. Patients felt they received personal feedback and that the nurse looked after them. The opportunity to communicate directly with a health care provider seems to be an important and highly valued feature across different patient populations. Our study suggests that this component may particularly appeal to people with high illness burden and low levels of social support.

In our study, high use of the advice section was associated with low social support and high illness burden among men with prostate cancer, but less so for women with breast cancer. This is in line with findings on prostate cancer patients’ preferences for informational support seen in studies of support groups [52,53]. Interestingly, no cluster of user characteristics were associated with high use of the forum. Although the forum was the component in which patients spent the most time, it seems that a discussion forum is not the place to turn to if one has little social support in addition to a high illness burden.

The finding that men with prostate cancer and women with breast cancer used WebChoice differently might indicate different needs for support and information. As described previously, low levels of social support without any other user characteristics was associated with high use of all components of WebChoice for women with breast cancer, but not for men with prostate cancer. Findings from a study of a computer support group for women with breast cancer were similar, showing a trend toward a higher volume of forum postings among those with lower levels of preexisting social support [33].

In this study, high levels of depression were associated with high use of several components in WebChoice among women with breast cancer whereas high levels of symptom distress were associated with high use among men with prostate cancer. One of the reasons could be difference in symptoms among prostate and breast cancer patients. For example, urinary incontinence and reduced sexual function are highly prevalent and bothersome for prostate cancer patients, potentially indicating different needs for support. It has been reported that men with prostate cancer use online support sites for information and women with breast cancer use them for emotional support [53,54]. The fact that low social support and high illness burden are associated with high use of several components, indicate that these characteristics are not barriers to use but rather function as motivators for use. The same pattern was seen previously in a study of an interactive cancer communication system [18].

### Differences Between Users and Nonusers

Consistent with earlier studies, previous computer experience made the patients somewhat more likely to use WebChoice compared with those with no or little former experience. As Internet access and computer literacy have increased in society, it suggests that more patients may be reached with Web-based support in the future.

Individuals without other illnesses in our study were more than twice as likely to use WebChoice compared to those with multiple illnesses. This finding is consistent with previous studies showing that users of Internet interventions are healthier than nonusers [22,55]. Chronically ill people are also reported to be less eHealth literate [32], thus they may not regard Web-based interventions as a suitable alternative for them, or they may be too ill to benefit. This could indicate that, at present, it may be more difficult to reach those with a higher illness burden. Another explanation for our findings might be that some of the WebChoice components (assessment, information, and advice) in this study specifically targeted breast and prostate cancer patients; thus, it could have been considered of limited value for patients with multiple conditions.

Known demographic predictors for use of Web-based interventions, such as education [20,21,24,26-28,56] and income [27,29], were not associated with use in our sample. This might be related to the inclusion criterion of having Internet access, and the fact that the sample was self-recruited. When the study started in 2006, 69% of the Norwegian population had access to the Internet [57]. This increased to 84% in 2008. Those with Internet access at that time were younger and had higher education and income compared to those without access. This is reflected in the study sample, as our participants were higher educated than the general population in Norway at that time. Interestingly, we did not find any statistically significant association between age and gender and frequency of use; other studies have described younger people and women as the most frequent users of Web-based interventions [20,24-26,28,29]. In our study we found a trend, although not statistically significant, that individuals over 50 years were almost twice as likely to be users compared to those under 50 (OR 1.75, 95% CI .74-4.13, *P*=.20), which corresponds to more recent studies in which older people are reported to be more motivated to use e-consultation than younger people [58], and also to use eHealth applications [22]. Moreover, age and diagnosis/gender were closely correlated in our study because all breast cancer patients were women and tended to be younger than the men with prostate cancer. Gender might also be a factor, but because breast and prostate cancer diagnoses are gender specific, it was not possible to distinguish between the effect of gender and diagnosis. As more people become computer literate, we might see several new groups of users of Web-based interventions.

The finding that only two-thirds of participants actively used WebChoice is consistent with other studies on the use of Web-based interventions, and it raises the question of what may motivate patients to use a system such as WebChoice. Interestingly, baseline levels of factors such as social support, symptom distress, and depression did not predict whether a patient became a user; the only variables that were associated with use were previous computer experience and having additional illnesses. This suggests that other factors may also be at play. Very few studies have investigated patients’ reasons for using or not using Web-based interventions, and previous studies on user experiences have primarily included active users only. Grimsbø et al [51] recently interviewed individuals with access to WebChoice to gain more insights into patients’ reasons and motivations for their use or nonuse. These interviews suggested that cancer patients had different needs, and that WebChoice was meaningful and suitable for some patients, but not all. Although some described perceived helpfulness as their main reason for using the application, others reported they did not want to assume a “sick” role or be reminded of having cancer and wished to “get on with their lives” as reasons for nonuse [51].

An important question is whether the factors that predict usage might also predict benefit. Similarly, does amount of usage relate to benefit? Higher dosage of an intervention has been connected to better outcomes of behavioral change programs for fruit consumption and maintenance of weight loss [21,59]. However, the dose of use and its relationship with effect is rarely reported in effect studies of Web-based interventions. Volume of use does not necessary lead to increased benefit. As in our study, different users utilized different components. Thus, the “right component” could be the one that also has some benefit, but not necessarily total volume of use, depending on the importance of the information or support provided. For example, reading advice for a very bothersome symptom once may be enough to learn how to relieve it and, thus, be tremendously beneficial, whereas reading postings on the discussion forum many times may help one to feel good, but may not be equally beneficial for managing symptoms. Reading advice once would be registered as low usage, engaging in the forum as high. As noted by some of the users of WebChoice [51], reading information on WebChoice could upset them or calm them down, and extensive use could be based on the fear of missing information about the cancer.

### Strengths and Limitations

The data from the server log provided detailed information on overall use of WebChoice and for each component on an individual and group level in this study, allowing us to perform the types of analysis presented here. Another strength is that we had baseline scores for all individuals and a low proportion of missing data. Data on use patterns of Web-based interventions can be challenging to analyze because they are rarely normally distributed, and there are often large variations in use. The use of LCA is a valid and valuable method used to analyze user patterns and identify subgroups of users according to specific characteristics, which adds to the strengths of this study.

However, several limitations also need to be addressed. First, participants in this study were self-selected and recruited through advertisement and pamphlets. In addition, they had to have Internet access. Increased willingness to participate in research studies is associated with higher education and use of the Internet (at least at the time of this study); therefore, these factors may limit generalizability. The men with prostate cancer in our study were younger (median 67 years) compared to patients diagnosed with prostate cancer in Norway in 2004 (median 72 years) [60]. Women with breast cancer in our study were also younger (median 50 years) compared to a study of 337 breast cancer patients in 2004 (median 55 years) [61]. Secondly, the relatively small sample put some constraints on the choice of statistical models. We chose to stratify all our statistical models by diagnosis/gender, thus making the comparison groups even smaller. In addition, the prostate cancer group was smaller than the breast cancer group, which might have further limited statistical power. The small sample size also reduced the number of variables that we were able to include in the LCA models. Given the limited statistical power, we chose to categorize our variables by using tertiles. We are aware that by doing so we might have lost some degree of information on differences in the underlying population. On the other hand, when fitting the models with categorical variables we were able to detect a direction on how low or high scores were associated with different use patterns of WebChoice. Computer experience and not having other illnesses were significantly related to increased probability of using WebChoice. However, given the size of our confidence intervals, reliability of our estimates may be somewhat uncertain. In addition, the measure of computer experience was based on a single question about the participant’s own opinion about their experience with computer use on a 5-item scale and those specific results should be interpreted with caution.

Furthermore, because this is an exploratory study with many tests of associations, other studies are warranted to replicate these findings. Finally, we do not know if the observed differences between breast and prostate cancer patients were related to the 2 diagnoses or to gender. Therefore, to clarify, future studies should include cancer diagnoses that affect both women and men.

### Conclusion

This study provides evidence about how different user characteristics influence the use of a Web-based illness self-management system among cancer patients. Such knowledge is crucial to target Web-based support systems to different patient groups. In our study, e-messages and advice for self-management support were components highly used by patients with low levels of social support and high levels of symptom distress and depression. Because patients with these characteristics may have a high need for support, these are components that may be particularly important to include in Web-based support systems for illness management support. Low levels of social support and high levels of depression influenced use of the system for women with breast cancer, whereas high levels of symptom distress influenced use for men with prostate cancer. Results highlight the importance of integration of multiple components in Web-based support systems to address different needs and reasons for use. LCA is a useful technique to identify subgroups of users and can be successfully applied for the analysis of user patterns of Web-based interventions. Our results will be employed in the further development of the WebChoice application, and can be utilized by developers and researchers in creation and evaluation of Web-based interventions optimizing content for different user groups.

## Multimedia Appendix 1

Data-driven tertile division of variables that is necessary for latent class analysis models.

## Multimedia Appendix 2

Supplemental tables depicting levels of single patient characteristics and associations with use of different components in WebChoice.

## Abbreviations

AIC: Akaike information criterion
BIC: Bayesian information criterion
CBI: Cancer Behavior Inventory
CES-D: Center for Epidemiological Studies Depression scale
CMIS: Comprehensive Model of Information Seeking
HRQOL: health-related quality of life
IQR: interquartile range
LCA: latent class analysis
MOS-SS: Medical Outcomes Study Social Support Survey
MSAS-SF: Memorial Symptom Assessment Scale-Short Form
OR: odds ratio
RCT: randomized controlled trial
