# Supplementary material for: How User Characteristics Affect Use Patterns in Web-Based Illness Management Support for Patients with Breast and Prostate Cancer
Source: J Med Internet Res. 2013 Mar 1;15(3):e34. doi: 10.2196/jmir.2285 (PMC3636230; doi:10.2196/jmir.2285)
Supplement: Supplementary file 1 [file jmir_v15i3e34_app1.pdf]

Multimedia Appendix 1

Data-driven tertile division of variables that is necessary for latent class analysis models

Variable		Scores	
	Low score/use	Medium score/use	High score/use
**Social support**	< 73	73-92	>92
**Depression**	< 7	7-15	> 15
**Symptom distress**	< 19	19-37	>37
**Quality of life**	< 0.8	0.8 - 0.9	>0.9
**Self efficacy**	< 205	205-239	>239
**Assessment duration**	< 8	8-23	>23
**Interventions duration**	< 9	9-26	>26
**Information duration**	< 16	16-45	> 45
**Message duration**	< 2	2-49	> 49
**Forum duration**	<34	34-206	> 206

N ranged between 47 and 57 for social support, depression, quality of life and self-efficacy scores
N ranged between 33 and 35 for the duration variables
Duration displayed in minutes

Distribution of breast and prostate cancer patients in the different tertiles

Variable	Scores				
	Breast cancer (N=56)		Prostate cancer (N=47)		
	n	%	n	%	*P*
**Social support**					.038
Low score	24	(43)	11	(23)	
Medium score	20	(36)	16	(34)	
High score	12	(21)	20	(43)	
**Depression**					.190
Low score	16	(29)	19	(41)	
Medium score	19	(34)	17	(37)	
High score	21	(38)	10	(22)	
**Symptom distress**					.324
Low score	16	(29)	19	(40)	
Medium score	16	(29)	14	(30)	
High score	24	(43)	14	(30)	
**Quality of life**					.468
Low score	16	(30)	15	(33)	
Medium score	25	(46)	16	(35)	
High score	13	(24)	15	(33)	
**Self efficacy**					.136
Low score	23	(42)	10	(25)	
Medium score	15	(27)	18	(45)	
High score	17	(31)	12	(30)	
**Assessment duration**					.820
Low use	17	(32)	17	(38)	
Medium use	17	(32)	14	(31)	
High use	19	(36)	14	(31)	
**Interventions duration**					.602
Low use	19	(34)	15	(33)	
Medium use	17	(30)	18	(39)	
High use	20	(36)	13	(28)	
**Information duration**					.211
Low use	17	(30)	17	(38)	
Medium use	16	(29)	17	(38)	
High use	23	(41)	11	(24)	
**Message duration**					.182
Low use	16	(29)	18	(39)	
Medium use	23	(41)	11	(24)	
High use	17	(30)	17	(37)	
**Forum duration**					<.001
Low use	9	(16)	25	(54)	
Medium use	24	(43)	10	(22)	
High use	23	(41)	11	(24)	

Total percent for each score/use is over 100 % in some cased due to round ofs.
