# Supplementary material for: How User Characteristics Affect Use Patterns in Web-Based Illness Management Support for Patients with Breast and Prostate Cancer
Source: J Med Internet Res. 2013 Mar 1;15(3):e34. doi: 10.2196/jmir.2285 (PMC3636230; doi:10.2196/jmir.2285)
Supplement: Supplementary file 2 [file jmir_v15i3e34_app2.pdf]

Multimedia appendix 2

Supplemental tables depicting levels of single patient characteristics and associations with use of different components in WebChoice.

Table 10: Levels of social support and association with use of assessment, advice and messages duration. The numbers represents item probabilities. All models were stratified by diagnosis and adjusted for age at inclusion.

	Latent class		
	1	2	3
**Prostate cancer**			
**Social support**			
Low	.14	.39	.14
Medium	**.65**[a]	.37	.19
High	.21	.24	**.67**
**Use of assessments**			
Low	.19	.31	**.51**
Medium	**.66**	.01	.45
High	.15	**.68**	.04
**Use of advice**			
Low	**.83**	.02	.36
Medium	.16	.32	**.54**
High	.01	**.65**	.09
**Use of messages**			
Low	**.56**	.20	.49
Medium	.01	.43	.17
High	.43	.37	.34
**Breast cancer**			
**Social support**			
Low	.11	**.63**	**.57**
Medium	.43	.26	.37
High	.46	.12	.05
**Use of assessments**			
Low	**.80**	.01	.19
Medium	.19	.11	**.64**
High	.01	**.88**	.18
**Use of advice**			
Low	**.95**	.01	.01
Medium	.04	.05	**.81**
High	.01	**.94**	.18
**Use of messages**			
Low	**.58**	.09	.16
Medium	.41	.25	**.55**
High	.01	**.66**	.29

[a] Items response probabilities >.5 in bold to facilitate interpretation
The most prominent class/classes is highlighted

Table 11: Levels of social support and association with use of advice, information and forum duration. The numbers represents item probabilities. All models were stratified by diagnosis and adjusted for age at inclusion.

	Latent class		
	1	2	3
		**Prostate cancer**	
**Social support**			
Low	.14	.20	.33
Medium	**.80**[a]	.30	.02
High	.06	**.50**	**.65**
**Use of advice**			
Low	.45	.43	.18
Medium	.34	.01	**.63**
High	.20	**.55**	.20
**Use of information**			
Low	.43	.03	**.54**
Medium	.48	.12	.45
High	.09	**.85**	.02
**Use of forum**			
Low	.40	.10	**.86**
Medium	.43	.11	.12
High	.17	**.79**	.03
		**Breast cancer**	
**Social support**			
Low	.06	**.58**	**.58**
Medium	.40	.33	.35
High	**.53**	.09	.07
**Use of advice**			
Low	**.98**	.01	.15
Medium	.01	.26	**.63**
High	.01	**.73**	.22
**Use of information**			
Low	**.53**	.01	.45
Medium	.23	.13	**.52**
High	.23	**.86**	.03
**Use of forum**			
Low	.09	.01	.42
Medium	**.67**	.20	.47
High	.24	**.79**	.10

[a]Items response probabilities >.5 in bold to facilitate interpretation
The most prominent class/classes is highlighted

Table 12: Levels of depression and associations with use of advice, messages and forum. The numbers represents item probabilities. All models were stratified by diagnosis and adjusted for age at inclusion.

		Latent class		
		1	2	3
		**Prostate cancer**		
**Depression**				
	High	.02	.20	.29
	Medium	**.76**[a]	.47	.17
	Low	.22	.33	**.54**
**Use of advice**				
	Low	**.62**	.25	.34
	Medium	.36	.36	.43
	High	.02	.39	.23
**Use of messages**				
	Low	**.95**	.01	**.69**
	Medium	.02	.39	.12
	High	.02	**.60**	.19
**Use of forum**				
	Low	**.56**	.15	**.98**
	Medium	.42	.36	.01
	High	.02	.49	.01
		**Breast cancer**		
**Depression**				
	High	.36	.01	**.60**
	Medium	.32	**.95**	.01
	Low	.32	.04	.39
**Use of advice**				
	Low	**.76**	.02	.01
	Medium	.22	**.54**	.28
	High	.03	.44	**.71**
**Use of messages**				
	Low	**.54**	.05	.11
	Medium	.46	**.60**	.24
	High	.01	.35	**.64**
**Use of forum**				
	Low	.17	.36	.04
	Medium	**.64**	.01	.41
	High	.19	**.62**	**.56**

[a]Items response probabilities >.5 in bold to facilitate interpretation
The most prominent class/classes is highlighted

Table 13: Levels of symptom distress and associations with use of assessment, advice and forum duration. The numbers represents item probabilities. All models were stratified by diagnosis and adjusted for age at inclusion.

	Latent class		
	1	2	3
		**Prostate cancer**	
**Symptom distress**			
High	.17	**.53**	.34
Medium	.30	.39	.14
Low	**.53**[a]	.08	**.52**
**Use of assessment**			
Low	**.58**	.13	.05
Medium	.37	.01	**.71**
High	.05	**.85**	.24
**Use of advice**			
Low	.34	.01	**.80**
Medium	**.56**	.14	.19
High	.10	**.84**	.01
**Use of forum**			
Low	**.78**	.27	.19
Medium	.21	.12	.41
High	.01	**.60**	.39
		**Breast cancer**	
**Symptom distress**			
High	.27	.36	**.59**
Medium	.42	.33	.15
Low	.31	.30	.25
**Use of assessment**			
Low	**.98**	.01	.24
Medium	.01	.15	**.72**
High	.01	**.85**	.03
**Use of advice**			
Low	**.98**	.01	.28
Medium	.02	.15	**.64**
High	.01	**.84**	.08
**Use of forum**			
Low	.02	.08	.34
Medium	**.87**	.23	.35
High	.11	**.70**	.30

[a]Items response probabilities >.5 in bold to facilitate interpretation
The most prominent class/classes is highlighted
